# Supplementary material for: Mercuric pollution of surface water, superficial sediments, Nile tilapia (Oreochromis nilotica Linnaeus 1758 [Cichlidae]) and yams (Dioscorea alata) in auriferous areas of Namukombe stream, Syanyonja, Busia, Uganda
Source: PeerJ. 2019 Oct 21;7:e7919. doi: 10.7717/peerj.7919 (PMC6812675; doi:10.7717/peerj.7919)
Supplement: Table S1 [file peerj-07-7919-s001.docx]

**Supplementary Table 1.** Recoveries from certified reference materials

| Certified reference material | Certified concentration ± standard error | Obtained value ± standard error | Recovery (%) |
| --- | --- | --- | --- |
| NRCCORMS-5 (elevated mercury in river water), National Research Council Canada, USA | 26.2 ± 1.3 pg/g | 26.1 ± 0.01 pg/g | 99.6 |
| CRM008-050, Resource  Technology Corporation, USA | 0.72 ± 0.03 μgg^-1^ (dry weight) | 0.74 ± 0.02 μgg^-1^ | 102.8 |
| SRM 2976 (freeze dried mussel tissue), National Institute of Standards and Technology, USA | 0.0610 ± 0.0036 μgg^-1^ (dry mass basis) | 0.06161 ± 0.01 μgg^-1^ | 101 |
